# Management of Multivessel Coronary Disease in ST-segment Elevation Myocardial Infarction

**DOI:** 10.1007/s11886-015-0632-6

**Published:** 2015-08-04

**Authors:** Amerjeet S. Banning, Anthony H. Gershlick

**Affiliations:** Department of Cardiovascular Sciences, University of Leicester Glenfield Hospital, Groby Road, Leicester, LE3 9QP UK; Department of Cardiology, University Hospitals of Leicester NHS Trust Glenfield Hospital, Groby Road, Leicester, LE3 9QP UK

**Keywords:** Myocardial infarction, Primary PCI, Multivessel disease, Complete revascularisation, Non-infarct-related artery, Revascularisation strategy

## Abstract

Primary PCI of infarct-related arteries is the preferred reperfusion strategy in patients presenting with ST-segment elevation myocardial infarction (STEMI). Up to 40 % of such patients demonstrate evidence of multivessel, non-infarct-related artery coronary disease. Previous non-randomised observational studies and their associated meta-analyses have suggested that in such cases only the culprit infarct-related artery (IRA) lesion should be treated. However, recent randomised controlled trials have demonstrated improved clinical outcomes with lower major adverse cardiovascular events (MACE) rates when complete revascularisation is undertaken either at index primary percutaneous coronary intervention (PPCI) or during index admission. These trials suggest that current guidelines pertaining to treatment of non-infarct-related artery (N-IRA) lesions in STEMI patients with multivessel disease may need to be reconsidered depending on future trials. However, issues remain around timing of N-IRA intervention, the use of fractional flow reserve (FFR) or intravascular imaging to guide intervention in N-IRA lesions and the need to demonstrate reductions in hard clinical endpoints (death and MI) after complete revascularisation; these issues will need to be addressed through future trials. Clinicians must judge on the currently available data, whether it is still safer to leave important stenosis in N-IRA untreated.

## Introduction

Primary percutaneous coronary intervention (PPCI) is the preferred reperfusion strategy in ST-segment elevation myocardial infarction (STEMI). It is estimated that 40 % of patients who present with STEMI have multivessel disease at index angiography [1–4]. How such significant lesions in the non-infarct-related vessels should be treated is controversial. There is evidence to suggest such patients have worse long-term outcomes compared to patients presenting with single-vessel disease, including a need for repeat revascularisation and repeat admissions with MI [5, 6]. However, undertaking PCI in by-stander lesions during STEMI can have potential complications. Current guidelines from the ACC/AHA [7] and ESC [8] recommend treatment of the culprit artery only in the absence of haemodynamic compromise.

The aim of this article is to review the current data pertaining to management of non-culprit lesions in patients presenting with ST elevation myocardial infarction, as well as some of the current guideline recommendations and the remaining outstanding questions regarding management of these lesions.

## Non-Randomised Studies and Current Guideline Recommendations

Much of the initial evidence base pertaining to complete revascularisation in STEMI patients with multivessel disease came from non-randomised observational cohort studies and registry data (Table 1; 9–19, 21–24). These observational studies contain some potential confounding factors, such as cardiogenic shock within the groups treated with complete revascularisation that may influence the outcome measures [24].

**Table 1:** Summary of non-randomised studies comparing complete and culprit-only revascularisation in STEMI patients

Study	Description	Exclusion criteria	Outcome measures.
Abe et al. 2012 [9]	Retrospective cohort study. 274 STEMI patients with multivessel disease undergoing culprit-only PCI (*n* = 220) or multivessel PCI (*n* = 54). Median follow-up of 374 days.		Increased risk of in-hospital death: 5.45 % vs 20.4 %, *P* < 0.05; all-cause death: 10.9 % vs 31.5 %, *P* < 0.05 and MACE: 27.7 % vs 46.2 %, *P* < 0.05, in patients undergoing multivessel PCI.
Cavender et al. 2009. [10]	Retrospective analysis of the NCDR database from 2004 to 2007 of patients undergoing primary PCI for STEMI. *N* = 28,936; 10.8 % underwent multivessel PCI (*n* = 3134).	Staged PCI treatment	Overall in-hospital mortality greater in patients with multivessel PCI (7.9 % vs. 5.1 %, *P* < 0.01). IN patients with STEMI and Cardiogenic shock, MV-PCI resulted in increased in-hospital mortality (36.5 % vs. 27.8 % for culprit-only, adjusted OR = 1.5, 95 % CI = 1.22–1.95)
Chen et al., 2005 [11]	Retrospective analysis of patients presenting with AMI (STEMI and NSTEMI) and evidence of multivessel disease. *N* = 1384; culprit-only PCI- *n* = 1189, multivessel PCI undertaken within 7 days of AMI, *n* = 239.	Pre-procedural cardiogenic shock, thrombolysis administered before PCI.	No difference in 1-year survival (MV-PCI = 0.93, 95 % CI = 0.87–0.95, culprit-only PCI = 0.92, 95 % CI = 0.92–0.95, *P* = 0.93) or 1 year survival from MI and revascularisation (MV-PCI = 0.78, 95 % CI = 0.73–0.84, culprit-only PCI = 0.78, 95 % CI = 0.75–0.81).
Corpus et al. 2004. [12]	Retrospective analysis of 506 patients presenting with STEMI and multivessel disease, undergoing culprit-only PCI (*n* = 354), PCI of IRA + N-IRA during same procedure (*n* = 26), PCI of IRA and N-IRA during index admission (*n* = 126).	PCI to SVG, LMS, acute occlusion after angioplasty or staged OP procedure.	MV-PCI associated with higher rate of reinfarction (13.0 % vs, 2.8 %, *P* < 0.001), revascularisation (25 % vs. 15 %, *P* = 0.007) and MACE (40 % vs 28 %, *P* = 0.006).
Dziewierz et al., 2010. [13]	Retrospective analysis of 1598 STEMI patients. 777 patients with MVD, 70 underwent MV-PCI, 707 culprit-only.		Higher rate of 30-day death, MI and revascularisation (adjusted OR = 1.33, 95 % CI = 0.57 = 3.10, *P* = 0.51), bleeding adjusted (OR = 0.80, 95 % CI = 0.32–2.02, *P* = 0.64) and 1 year death (adjusted OR = 2.04, 95 % CI = 0.89–4.66, *P* = 0.09). Not statistically significant.
Han et al., 2008. [14]	*N* = 242. Retrospective analysis. 192 patients underwent PCI to IRA-only, 93 patients underwent N-IRA PCI 7–15 days after PPCI to IRA.	Cardiogenic shock, LMS, pulmonary oedema, LV rupture	No significant difference in 12-month MACE (11.5 % vs 15.1 %, *P* > 0.05) or target lesion revascularisation (8.1 % vs 7.6 %, *P* > 0.05). Higher rates of recurrent angina in IRA-only group (10.1 % vs 2.1 %, *P* < 0.05).
Hannan et al., 2010 [15]	Registry of 3251 patients undergoing either culprit-only PCI, PCI at time of index procedure and staged (within 60 day) PCI to N-IRA lesions. Propensity-matched analysis.	Shock, previous heart surgery, LMS disease, thrombolysis	Non-significant trend towards higher in-hospital, 12- and 42-month mortality in MV-PCI undertaken at time of index procedure compared with culprit-only PCI. Significant in-hospital mortality excess with index procedure MV-PCI compared to culprit-only PCI when haemodynamic instability excluded. Lower 12-month mortality with staged MV-PCI compared to culprit-only PCI (1.3 % vs 3.3 %, *P* = 0.04).
Khattab et al., 2007. [16]	Prospective study of 73 STEMI patients, with MV-PCI undertaken in the first 28 patients and culprit-only PCI with either planned staged or ischaemia-driven PCI of N-IRA lesions in 45 patients.		Similar baseline characteristics. MACE (death, MI and TVR) at 12 months similar between the two treatment strategies (MV-PCI = 24 %, culprit-only = 28 %, *P* = 0.73). No difference in revascularisation (MV-PCI = 24 %, culprit-only PCI = 28 %, *P* = 0.73).
Kornowski et al., 2011. [17]	Analysis of 668 STEMI patients from the HORIZONS-AMI trial, undergoing either same-setting MV-PCI (*n* = 275) or staged PCI (*n* = 373). NO difference in baseline characteristics between groups, except higher rates of TIMI0/1 flow in culprit vessel in staged PCI group.	As per HORIZONS-AMI trial: Prior thrombolysis, bivalirudin, GPI, LMWH, warfarin, bleeding diathesis, transfusion.	Higher rates of 1-year mortality, cardiac mortality and definite/probable stent thrombosis in patients undergoing same-setting MV-PCI compared to staged PCI. Single-setting MV-PCI was independently predictive of 30-day and 1-year mortality and MACE (death, reinfarction, TVR and stroke).
Lee et al., 2012 [18]	1644 STEMI patients from Korean registry with multivessel disease undergoing culprit-only (*n* = 1106) or multivessel PCI (*n* = 538). Lower incidence of 3vD in culprit-only group, higher number of patients with EF <40 % in culprit-only group.		No significant difference in MACE (all-cause death, MI, revascularisation, CABG) at 30 days or 12 months between the two groups. Higher rate of TLR in MV-PCI group (2.4 % culprit-only, 5.9 % MV-PCI, *P* < 0.0001).
Manari et al. 2014. [19]	2061 STEMI patients with multivessel disease undergoing PPCI; 706 culprit-only, 367 multivessel index procedure, 988 staged PCI at 60 days.	Shock, CTO in one vessel, severe LMS, previous CABG	Multivariate analysis showed higher rates of 30-day and 2-year mortality in culprit-only PPCI compared to staged MV-PCI. Short-term mortality rates higher for multivessel PCI compared to culprit PPCI.
Quarani et al. 2008. [20]	Prospective non-randomised study of 120 consecutive patient presenting with STEMI and multivessel disease underwent either culprit-only PCI (*n* = 25) or complete revascularisation (*n* = 95).	Cardiogenic shock, LMS >50 %.	Reduction in in-hospital MACE events (in-hospital mortality, recurrent ischaemia, reinfarction, acute heart failure) with complete revascularisation (16.7 % vs 52 %, *P* = 0.001), driven by reductions in recurrent ischaemia, reinfarction and acute heart failure. Increased CIN seen in MV-PCI group.
Rigattieri et al., 2007. [21].	Retrospective comparison of STEMI patients with multivessel PCI undergoing culprit-only (*n* = 46) or early, staged N-IRA PCI (*n* = 64).	Cardiogenic shock, LMS disease, severe valvular disease, previous CABG.	Non-significantly Higher in-hospital MACE with complete revascularisation (20.3 % vs. 10.8 %, *P* = 0.186), driven by periprocedural MI. Lower follow-up MACE events with staged MV-PCI.
Roe et al., 2001. [22].	Retrospective study of 68 cases of multivessel PCI at time of IRA-primary PCI, matched to 61 cases of primary PCI IRA-only PCI.		IN the primary PCI subgroup analysis: MACE higher in MV-PCI (35.3 % vs. 27.9 %, *P* = 0.63). Significantly higher risk of stroke with MV-PCI (10.3 % vs 0 %, *P* = 0.01) and non-significantly increase 6-month mortality and reinfarction.
Toma et al. 2010 [23].	Retrospective analysis of 2201 STEMI patients with MVD in the APEX-AMI trial; 217 underwent N-IRA PCI, 1984 underwent IRA-PCI alone.		90-day death rate significantly higher in N-IRA PCI group (12.5 % N-IRA; 5.6 % IRA-only, *P* (log rank) <0.001). 90-day composite of 90-day death, CHF and shock from randomisation higher in N-IRA PCI group.
Varani et al., 2008. [24].	Retrospective study of 399 patients with STEMI and MVD (IRA-only = 156, MV-PCI at index procedure = 147, MV-PCI staged pre discharge = 96).		After exclusion of patients with cardiogenic shock and pulmonary oedema (seen more often in index procedure MV-PCI); MV-PCI 30 day mortality = 2.8 %, IRA-only = 6.3 %. Rate of MV-PCI similar to STEMI patients with single-vessel disease in this cohort. No difference in 30-day mortality between single-setting and staged procedure for N-IRA lesion

Abbreviations: *MV-PCI* multivessel percutaneous coronary intervention, *MACE* major adverse cardiovascular events (as defined in each study), *LMS* left main stem, *IRA* infarct-related artery, *N-IRA* non-infarct-related artery, *MI* myocardial infarction, *STEMI* ST elevation myocardial infarction, *CTO* chronic total occlusion, *SVG* saphenous vein graft, *MVD* multivessel disease

A meta-analysis of early observational and randomised studies [25•] showed benefit in longer-term mortality with complete revascularisation if non-infarct-related artery (N-IRA) PCI is performed on a staged basis as opposed to being undertaken the time of the index PCI.

A separate meta-analysis by Bangalore et al. included 19 studies (2 randomised studies, 17 observational or registry studies) to evaluate early (<30 days) and long-term safety and efficacy of multivessel PCI [26]. Of the 61,764 patients included, multivessel PCI was performed in only 9690 (16 %) of patients with the remaining 84 % undergoing culprit lesion only PCI. Overall there was no significant difference in 30-day mortality (OR = 1.04, 95 % CI = 0.93–1.15); however, when stratified by timing of intervention, 30-day mortality was lower with the complete revascularisation when N-IRA PCI was performed as a staged procedure (OR = 0.44, 95 % CI = 0.33–0.59), while there was a trend towards worse 30-day mortality if N-IRA PCI was performed at the same time as the culprit PPCI (OR = 1.19, 95 % CI = 1.06–1.34). Longer-term mortality (>1 year post-primary PCI) was significantly lower with complete revascularisation (OR = 0.67, 95 % CI = 0.58–0.79) and again, this effect was attenuated if complete revascularisation was undertaken at the same sitting as primary PCI of the culprit lesion (OR = 0.91, 95 % CI = 0.73–1.14). A 30-day and longer-term major adverse cardiovascular event (MACE) was significantly lower with complete revascularisation regardless of when the N-IRA PCI was undertaken. There was a high degree of heterogeneity seen amongst the studies within this meta-analysis, particularly in those where complete revascularisation was undertaken at the same sitting.

A propensity-matched analysis of 3984 patients with STEMI and multivessel disease presenting in eight cardiac centres within London between 2004 and 2011 looked at outcomes from culprit-only and complete revascularisation at time of PPCI [27]. Cardiogenic shock and LMS disease was excluded from the analysis. In this cohort, 555 patients, i.e. 13.9 % of patients with multivessel disease, underwent multivessel PCI. Although propensity-matched analysis suggested an improved survival with culprit-only PCI during the index procedure (HR = 0.64; 95 % CI = 0.45–0.90; *P* = 0.010) and reduced MACE with culprit-only PCI (HR = 0.49; 95 % CI = 0.32–0.76; *P* = 0.002), there are some important limitations to this study. Specifically, it could not be ascertained how many patients in the culprit-only cohort went on to have staged complete revascularisation. Secondly, presence of cardiogenic shock was based on use of inotropes and IABP in cases as no haemodynamic data were available, hence it is possible a proportion of the patients undergoing multivessel disease had early cardiogenic shock prior to receiving complete revascularisation.

The question has been raised as to the potential downsides of leaving a non-infarct-related artery untreated. In a retrospective analysis of 28,282 STEMI patients, it was shown that the presence of multivessel disease (52.8 %) appeared to confer a worse prognosis, with the presence of N-IRA disease being significantly associated with 30-day mortality (unadjusted, 4.3 vs 1.7 %, respectively; risk difference, 2.7 %[95 % CI = 2.3–3.0 %], *P* < .001; and adjusted, 3.3 vs 1.9 %, respectively; risk difference, 1.4 % [95 % CI = 1.0–1.8 %], *P* < .001) [28].

The 2012 ESC guidelines on STEMI management recommend “PCI should be limited to the culprit vessel with the exception of the presence of cardiogenic shock and persistent ischaemia after PCI of the supposed culprit lesion” (Class IIa recommendation, Level of evidence B) [7]. Prior to the publication of recent randomised studies, in 2013 the ACCF/AHA guidelines give PCI to an non-infarct-related artery at the time of primary PCI without haemodynamic instability a class III recommendation (Level of evidence B) [8]. These recommendations are largely drawn on the preceding non-randomised studies and meta-analyses as outlined above.

## Randomised Studies

Two recently reported UK-based RCTs comparing culprit-only revascularisation to treatment of any trial-defined significant additional non-IRA in STEMI with multivessel disease have opened a debate as to how these patients might be managed.

PRAMI was a randomised open trial that recruited 465 patients over 4 years, who presenting with STEMI were found to have multivessel coronary disease [29••]. The study defined the presence of multivessel disease as a non-infarct-related artery (N-IRA) lesion >50 % stenosis within a major epicardial artery. Patients recruited to the study were randomised to receive either culprit-only PCI or PCI of both culprit and N-IRA lesions at the same sitting (i.e. during index primary PCI procedure). The primary outcome measure was a composite of cardiovascular death, new MI and refractory angina defined as angina despite medical therapy supported by evidence of objective ischaemia The study was discontinued early by the DSMB at a mean follow-up of 23 months, due to a significant difference in primary endpoint in favour of complete revascularisation (HR = 0.35, 95 % CI = 0.21–0.58, *P* < 0.001) having been reached. In particular, this appeared to be driven primarily by a significant reduction in refractory angina (HR = 0.35, 95 % CI = 0.13–0.75) and non-fatal myocardial infarction (HR = 0.32, 95 % CI = 0.13–0.75), although no significant reduction in mortality was shown. The randomisation process was not stratified for timing of intervention in relation to symptom onset, nor for site of infarction. Indeed, the infarct-related artery (IRA)-only group in this study demonstrated a higher proportion of diabetic patients and those admitted with anterior MI, both known to confer worse prognosis following acute myocardial infarction.

CvLPRIT was a second open randomised trial comparing culprit lesion only with complete intervention in patients presenting with STEMI and evidence of multivessel disease [30••]. N-IRA lesions were defined as those being >70 % diameter stenosis. A total of 296 patients were recruited to the study over a similar timeframe as PRAMI. Patients were randomised to either complete or culprit-only revascularisation following confirmation on index angiography of one or more N-IRA lesions. In this study, the N-IRA PCI could be undertaken either at the time of index primary PCI to the culprit, or on a separate occasion but within the same admission. Like PRAMI, this trial showed a reduction in the primary endpoint of death, MI, heart failure and ischaemic-driven revascularisation with complete revascularisation (HR = 0.45, 95 % CI = 0.24–0.84, *P* = 0.009). Within the components of the primary endpoint, while showing a trend towards lower adverse events with complete revascularisation, none of the individual components of the primary endpoint attained statistical significance. In addition, there was a very early separation of the Kaplan-Meier event curves between the two randomised groups, suggesting an early benefit with complete revascularisation. As with PRAMI, the trial had small number of events in both arms, and the open-label nature of the study may have an impact on revascularisation in patients with known untreated N-IRA lesions in the culprit-only group.

Subsequent important contributions to the evidence base include the Third DANish Study of Optimal Acute Treatment of Patients with ST-segment Elevation Myocardial Infarction. The PRImary PCI in MULTIvessel Disease (DANAMI3-PRIMULTI) trial [31] recently reported and was a randomised trial recruiting 627 patients from a cohort of 2239 patients who had initially been randomised to the “ischaemic post-conditioning (iPOST)” or “deferred stenting (DEFER)” other arms of the study, and in whom there was evidence of multivessel disease with successful primary PCI of the IRA. The patients were then randomised to receive either fractional flow reserve (FFR)-guided intervention of N-IRA lesions, or medical management with no further intervention. The primary endpoint was composite of all-cause mortality, non-fatal myocardial infarction and ischaemic-driven revascularisation of N-IRA lesions. As with CvLPRIT and PRAMI, this study also showed benefit in favour of complete revascularisation (HR = 0.56, 95 % CI = 0.38–0.83, *P* = 0.004); however, there was no significant difference in all-cause mortality nor in non-fatal myocardial infarction between the two groups the reduction in composite endpoint being primarily driven by a reduced rate of ischaemic-driven revascularisation in the FFR-guided complete revascularisation group (HR = 0.31, 95 % CI = 0.18–0.53, *P* < 0.001).

PRAGUE-13 randomised 214 STEMI patients with multivessel disease to either complete revascularisation between 3 and 40 days post PPCI or IRA-PCI only [32]. This trial demonstrated no difference in MACE (death/MI/CVA) between the two groups (HR = 1.36, 95 % CI = 0.66–2.74). However, the severity of N-IRA lesions was less than in other studies, with only 6.1 % of N-IRA lesions being classed as >95 % stenosis. This study did not include ischaemia-driven revascularisation as part of the endpoint and was underpowered to detect differences in hard endpoints. It is generally regarded as a trial of less significant non-IRA lesions.

In 2014, PRAMI and CvLPRIT led to the ACC withdrawing a “do-not-do” recommendation of complete revascularisation in STEMI patients with multivessel disease. The results of these trials and associate meta-analyses suggest that the current guideline recommendations pertaining to management of multivessel disease in STEMI should be revisited.

## Safety of Complete Revascularisation

Performing complete revascularisation has been shown to result in greater use of contrast, prolonged procedural time and increased exposure to radiation especially when performed at the same time as the index primary PCI for the culprit lesion [29••, 30••, 31, 33]. In spite of this, a pooled analysis of PRAMI, CvLPRIT and the trial by Politi et al. has shown no increase in CVA, bleeding or contrast-induced nephropathy [34•]. Thus, the data suggest, although procedure times and contrast use may be increased with complete revascularisation, this does not translate to an increased risk of adverse events. Similarly, there was no difference in rates of periprocedural MI, stroke, contrast-induced nephropathy or bleeding between the two revascularisation strategies in the DANAMI3-PRIMULTI study [31].

## Is There a Prognostic Benefit for Complete Revascularisation?

Although the recent RCTs demonstrate a benefit of composite MACE endpoints with complete revascularisation, in CvLPRIT and DANAMI3-PRIMULTI, this has mainly been driven by a reduction in ischaemia-driven revascularisation within the complete revascularisation arm. In PRAMI, a reduction in refractory angina and non-fatal myocardial infarction was seen; however, ischaemia-driven revascularisation (in this study as a secondary endpoint) was also reduced. However, none of these trials were adequately powered to detect a difference in prognostic clinical endpoints of death or myocardial infarction.

A meta-analysis of four RCTs that compared culprit-only and complete revascularisation (CvLPRIT, PRAMI, Politi and HELP-AMI) consisting of 1044 randomised patients demonstrated significant reduction in long-term (≥1 year) all-cause mortality (RR: 0.57, 95 % CI = 0.36–0.92, *P* = 0.02), cardiovascular death (RR: 0.38, 95 % CI = 0.20–0.73, *P* = 0.004) and myocardial infarction (RR: 0.41, 95 % CI = 0.23–0.75; *P* = 0.004) with complete revascularisation compared to culprit-only PCI. In all three analyses, there was a low degree of heterogeneity between the included studies [34•]. There was a low degree of heterogeneity between the included studies (*I*^2^ = 0 % for all three outcome measure comparisons).

Similarly, meta-analyses described below including both randomised trials and non-randomised studies have demonstrated a reduction in long-term mortality rates when complete revascularisation is performed as a staged procedure (i.e. not at the index primary PCI procedure) [25•, 35].

While the above meta-analyses indicate a signal towards improved death and MI with complete revascularisation, they are limited by the design limitations of the individual studies and also the heterogeneity between the studies in terms of inclusion and exclusion criteria, timing of the N-IRA procedures and outcome measures. Hence, this question can only be answered robustly by appropriately powered randomised trials with sufficient numbers to detect any differences in death and MI between strategies.

## Timing of Complete Revascularisation

An issue not fully addressed by the recent randomised trials is the optimal timing of complete revascularisation. Mechanistically, performing complete revascularisation at the time of culprit primary PCI could potentially lead to extension of infarcted muscle if the attempted PCI to the N-IRA were to be complicated by coronary dissection or stent thrombosis, so-called double jeopardy.

Potential timing for complete revascularisation include undertaking it at the time of index primary PCI, as a separate procedure during the index admission, or as a planned elective outpatient procedure for the N-IRA lesions.

Hannan et al. (2010) published registry data of multivessel disease in STEMI undergoing primary PCI between Jan 2003 and June 2006 [15]. A total of 3521 patients were included, of whom 259 underwent staged PCI during the index admission, and 538 underwent staged intervention of N-IRA lesions within 60 days of discharge from index admission. Patients with shock, previous heart surgery, LMS disease or thrombolysis prior to PCI were excluded. Propensity matching was undertaken for comparison of inpatient, mortality between culprit-only PCI, complete revascularisation during index procedure and staged complete revascularisation as defined above.

This study showed in patients without features of haemodynamic instability, increased in-hospital mortality occurred with complete revascularisation at index procedure compared to culprit-only (complete revascularisation at time of index PCI = 2.4 %, culprit-only PCI = 0.9 %, *P* = 0.04).

There was however a difference in 12-month mortality in favour of complete revascularisation when undertaken as a staged procedure within 60 days compared to culprit-only PCI (staged complete revascularisation = 1.3 %, culprit-only PCI = 3.3 %, *P* = 0.04). Overall, the findings from this registry data indicate that a potential mortality benefit was present if N-IRA PCI was performed as a staged procedure and also suggested potentially worse outcomes by complete revascularisation at the time of the index procedure.

A meta-analysis of randomised and non-randomised studies [25•] suggests that index procedure complete revascularisation may be associated with increased in-hospital morality, while performing staged complete revascularisation was associated with reduced in-hospital mortality (OR 0.35, 95 % CI = 0.21–0.59; *P* < .001; *P* interaction <.001). The same meta-analysis also showed reduced longer-term mortality with complete revascularisation (OR 0.74, 95 % CI = 0.65–0.85, *P* < .001) and repeat PCI (OR 0.65; 95 % CI = 0.46–0.90, *P* = .01 [randomised OR 0.31, 95 % CI = 0.17–0.57, *P* < .001; non-randomised OR 0.88, 95 % CI = 0.59–1.31, *P* = .54]).

A paired and network meta-analysis of 14 studies including 40,280 patients, compared three timing strategies in STEMI patients with multivessel disease; staged PCI, (defined in this analysis as separate procedure either during index admission or within 1 month of the primary PCI), complete revascularisation during index primary PCI procedure, and culprit-only PCI. This analysis showed lowest short- and long-term mortality rates in patients undergoing staged complete revascularisation [35]. The majority of the studies within this analysis were retrospective cohort studies, and although in some cardiogenic shock were excluded, given that for retrospective studies the decision to perform either index procedure or staged complete revascularisation was not prospectively registered, the analysis could not fully adjust for any important patient characteristics that may have influenced the decision for index procedure complete revascularisation.

While the above studies suggest deferring N-IRA PCI to a staged procedure, data from the randomised studies with regard to timing of the intervention do not support this strategy. The Politi study [33] randomised patients to receive either inpatient, staged outpatient complete revascularisation or culprit-only revascularisation. This showed lower rates of MACE with complete revascularisation compared to culprit-only; however, there appeared to be no difference in MACE event rates between inpatient and staged outpatient complete revascularisation.

The CvLPRIT study suggested that there may be benefit from early intervention, with a landmark analysis of the data at 30 days showing a trend towards improved early MACE reduction with complete revascularisation (HR = 0.45, 95 % CI = 0.19–1.04, *P* = 0.055). Although this did not achieve statistical significance, there is a clear early separation in the Kaplan-Meier curves for MACE. A subsequent analysis of the 139 patients who underwent complete revascularisation within the study showed no difference in the 12-month primary composite MACE endpoint (death/MI/HF/repeat revascularisation) between complete revascularisation at the index procedure compared with staged inpatient revascularisation (6.2 % index complete vs. 11.9 % staged inpatient; HR = 0.51, 95 % CI = 0.16–1.67, *P* = 0.29). Although when looking at composite of death/MI/HF, there was a non-significant trend towards lower rates with index procedure complete revascularisation (3.1 vs. 11.9 %; HR = 0.26, 95 % CI = 0.06–1.08, *P* = 0.06). These post-hoc analysis results have to be interpreted with caution as the patients were not randomised to performing either index procedure or staged inpatient complete revascularisation. .

Comparison of index procedure and staged outpatient complete revascularisation was also examined in a randomised trial by Ochala et al. [36]. In this study, 98 patients were randomised to receive either one-stage (index procedure) or two-stage (elective outpatient) complete revascularisation. Cardiogenic shock and LMS >50 % were excluded from the study. There was no difference in MACE (defined as all-cause death, MI, urgent revascularisation) or improvement in LVEF between the 2 groups, with staged revascularisation of N-IRA lesions performed 27.3 ± 12.8 days after primary PCI.

When N-IRA PCI should be performed remains an issue for further research in adequately powered studies.

## Assessing the Significance of Non-culprit Lesions: Functional vs. Anatomical vs. Plaque Composition

Most of the observational and randomised studies have used angiographic diameter stenosis severity to determine N-IRA lesions requiring treatment. However, it is known within the context of stable angina assessment that determining the haemodynamic significance of lesions using fractional flow reserve may allow for more accurate identification of lesions requiring intervention.

Ntaliansis et al. showed that amongst 75 STEMI patients, there was no significant change in the FFR measurement in the non-IRA when repeated at a mean of 35 ± 4 days post initial procedure (0.78 ± 0.10 vs. 0.76 ± 0.10, *P* = NS) [37].

Preliminary data from the COMPARE-ACUTE study also suggest a disparity between angiographic-determined significance and haemodynamically significant N-IRA lesions in STEMI patients. In 408 STEMI patients with evidence of multivessel disease defined by presence of N-IRA lesions with diameter stenosis of >50 %, FFR measurements performed at the time of index procedure showed that in 56.5 % the FFR was negative (<0.8) [38].

Dambrink et al. randomised 121 STEMI patients with multivessel disease (N-IRA lesions of >50 % diameter stenosis) in a 2:1 fashion to receive either early FFR-guided revascularisation of N-IRA lesions or to conservative medical treatment [39]. Randomisation occurred following successful primary PCI of the culprit lesion. Repeat angiography in the invasive group was performed at a median of 7.5 days after primary PCI.

Of the 80 patients allocated to the invasive treatment arm, only 65 underwent FFR assessment of non-culprit lesions. From these, 42 patients (50 lesions) demonstrated positive FFR measurement, with 23 patients (41 lesions) with negative FFR and hence managed conservatively.

At 6 months, there was no difference in MACE rates (death/MI/repeat PCI) between the two strategies using an intention-to-treat analysis (16 vs. 22 %, *P* = 0.292). A pre-specified per protocol analysis showed significantly higher rates of repeat PCI in the culprit-only group (6 vs. 22 %, *P* = 0.017), with death and MI occurring solely in the FFR-guided revascularisation group, leading to comparable 6-month composite MACE rates (14 vs. 22 %, *P* = 0.292). A longer-term follow-up to 3 years of these patients showed no significant difference in all-cause mortality between invasive and conservatively managed groups, higher rate of death and non-fatal MI with the invasively treated group (13.4 % all in the invasive group, *P* = 0.002); however, lower rate of PCI in non-culprit vessel in the invasive group (8.9 vs. 32.5 %, *P* = 0.001). There was no difference in MACE between the two groups (35 vs. 35 %, *P* = 0.896) [40]; however, the study was not powered to detect difference in MACE.

DANAMI3-PRIMULTI also employed FFR as a means of selecting N-IRA lesions for intervention. There was a significant reduction in MACE event rates with complete revascularisation, driven mainly by a reduction in ischaemia-driven revascularisation in the complete revascularisation arm [31]. However, no assessment was made of the haemodynamic significance of N-IRA lesions in the medically managed group.

None of the above studies addresses the question of whether FFR provides a more sensitive means of determining which N-IRA lesions should be intervened upon compared to assessment by angiographic significance. The COMPARE-ACUTE trial (NCT01399736) is currently ongoing and will recruit 885 patients, comparing FFR-guided complete revascularisation with culprit-only PCI. This study will also compare MACE outcomes as one of the subgroup analyses staged PCI to N-IRA lesions that are angiographically significant (DS >50 %) but with a negative FFR with not intervening on these lesions.

While clearly there is evidence to support use of FFR in determining significant flow-limiting lesions in stable angina, one aspect with regard to N-IRA lesions in STEMI is that they may represent a more vulnerable plaque morphology compared to stable angina patients, and hence outcomes may not solely be driven by the haemodynamic significance of the lesion. Analysis non-culprit lesions of ACS patients using VH-IVUS has shown a greater proportion of vulnerable plaques, with greater proportion of necrotic core and thin-cap fibroatheroma compared with stable coronary lesions [41]. TCFA lesions were also seen in 46.7 % of non-culprit lesions within ACS population from the PROSPECT study; logistic regression demonstrated the presence of TCFA at non-culprit site was an independent predictor of future MACE events [42].

Similarly, Kato et al. [43•] have demonstrated differences in plaque composition in non-culprit lesions from ACS patients compared to stable angina plaques using three-vessel OCT. Specifically, non-culprit plaques demonstrated wider lipid arc (147.3 ± 29.5° versus 116.2 ± 33.7°, *P* < 0.001), a longer lipid length (10.7 ± 5.9 versus 7.0 ± 3.7 mm, *P* = 0.002), a larger lipid volume index [averaged lipid arc × lipid length] (1605.5 ± 1013.1 versus 853.4 ± 570.8, *P* < 0.001) and a thinner fibrous cap (70.2 ± 20.2 versus 103.3 ± 46.8 μm, *P* < 0.001). Also, there was a higher prevalence of TCFA in non-culprit ACS lesions compared to stable angina patients. These findings suggest that non-culprit plaques in ACS patients are inherently more vulnerable and may account for recurrent ischaemic events occurring after the primary event at these sites.

Hence, one could hypothesise that in the context of STEMI and multivessel disease, plaque morphology in addition to haemodynamic significance, should drive decision making in terms of N-IRA PCI. Further studies using VH-IVUS or OCT to determine plaque composition in N-IRA lesions and impact of this on preventing future MACE events, particularly hard endpoint of non-fatal MI, would be required. Findings from intracoronary near infra-red spectroscopy have identified plaques with large lipid cores at culprit sites for STEMI and NSTEMI [44, 45]. This may also provide a useful tool for site-specific identification of N-IRA plaques that may give rise to future coronary events; however, this hypothesis would also require validation in large prospective randomised studies.

## Conclusions

The recently reported PRAMI, CvLPRIT and DANAMI3-PRIMULTI randomised controlled trials have shown that in patients presenting with STEMI and evidence of additional non-infarct-related artery lesions, a strategy of complete revascularisation results appears to result in improved clinical outcomes compared with culprit-only PCI. These studies shed new light on the management of such patients, as previous studies were non-randomised and retrospective in nature. In addition, these RCTs reflect more contemporary clinical practice with regard to use of drug-eluting stents, radial access as the preferred vascular approach and use of newer generation P_2_Y_12_ inhibitors such as Prasugrel and Ticagrelor, all of which may add towards the improved clinical outcomes seen compared to previous trials.

There however remain unanswered questions with regard to the optimal management strategy of STEMI patients with multivessel disease. The timing of intervention in N-IRA arteries remains unclear, with current data presenting conflicting evidence in terms of whether same sitting, same admission or staged procedures would result in improved outcomes. Another issue is the optimal method of determining which N-IRA lesions require intervention, either by angiographic severity or use of FFR to guide intervention based on haemodynamic significance of N-IRA lesions.

Finally, although the recently reported RCTs demonstrate improved MACE outcomes with complete revascularisation, these are mostly driven by ischaemic-driven revascularisation and MI in the case of PRAMI. Hence, larger studies powered to detect differences in hard clinical endpoints of death and MI are required.
